# An Illustrative Case of Cervical Spondylotic Myelopathy and Medullary Cavernoma: Which Should Be Treated First?

**DOI:** 10.7759/cureus.70378

**Published:** 2024-09-28

**Authors:** Marco Antonio Munuzuri-Camacho, Michel Gustavo Mondragon Soto, Domingo J Coutinho Thomas, Obet Canela-Calderon, Jorge Del Pino-Camposeco, Eliezer Villanueva-Castro, Juan N Arriada-Mendicoa, Juan Antonio Ponce-Gómez

**Affiliations:** 1 Department of Neurosurgery, Instituto Nacional de Neurología y Neurocirugía Manuel Velasco Suárez, Mexico City, MEX

**Keywords:** case study, cervical spine, cervical stenosis, degenerative spondylotic myelopathy, medullary cavernoma

## Abstract

Cervical spondylotic myelopathy (CSM) and spinal cavernoma (SC) represent distinct yet challenging conditions. CSM manifests as progressive neurological dysfunction, whereas SC denotes a benign vascular lesion. The need for documented cases featuring CSM and SC highlights the absence of evidence-based management guidelines for such scenarios.

CSM typically presents as a gradual neurological decline, predominantly afflicting middle-aged men. Pathophysiological mechanisms involve axonal stretching and ischemia. Classic symptoms encompass gait instability, bladder dysfunction, limb paresis, hyperreflexia, and somatic pain. Diagnostic complexities persist due to the weak correlation between radiological findings and clinical severity, complicating treatment decisions.

In contrast, SC, although often asymptomatic, exhibits a preference for the thoracic and cervical segments and can mimic intramedullary tumors. Diagnosis typically relies on magnetic resonance imaging (MRI) due to the limitations of angiography.

This case study of a 66-year-old male with concurrent CSM and SC sheds light on the diagnostic and treatment challenges encountered. Surgical intervention targeting CSM preceded SC resection, resulting in significant clinical improvement.

In conclusion, managing patients with concurrent CSM and SC necessitates a tailored approach, considering each condition's distinct characteristics and treatment goals. Further research is warranted to establish standardized management algorithms for this complex clinical scenario.

## Introduction

Cervical spondylotic myelopathy (CSM) is the compression of the spine due to a degenerative phenomenon in the cervical spine, causing gradual and progressive neurological dysfunction [1]. Many cases are associated with facet arthropathy, spondylosis, disc degeneration, subluxation of vertebral bodies, hypertrophy, ossification, or calcification of supporting ligaments [2]. As the most common cause of spinal dysfunction, its prevalence is estimated to be 2.3% worldwide. However, it is probably underestimated due to its degenerative conditions and the expansion of the global population [3]. It predominantly affects men at 66%, with an average age of 56 [4]. Its pathophysiological mechanism involves lesions associated with axonal stretching and ischemia of the spinal cord due to compression. The most commonly affected level is C5-C6 [5]. The clinical picture is insidious, with classic symptoms including gait instability, dysfunction of bladder automation, limb paresis, hyperreflexia, and altered proprioception with varying degrees of somatic pain [6].

On the other hand, an intramedullary spinal cord cavernoma (ISCC) is a neoplastic vascular spinal lesion considered angiographically silent. Histopathologically, it is defined as benign vascular hamartoma composed of distinct thick- and thin-walled sinusoid vascular channels deprived of normal neural parenchymal tissue, feeding arteries, or large daring veins. ISCCs make up 20% of intramedullary tumors, but their simultaneous presentation with spondylotic degenerative cervical disease is infrequent. Cases, where both conditions coexist, could be complex due to the overlapping symptomatology and challenges in treatment approaches. ISCCs may require surgical intervention, especially if they bleed or cause significant neurological deficits. The management of degenerative spondylotic myelopathy may involve both conservative treatments, such as physical therapy and medications to relieve symptoms, and surgical options to decompress the spinal cord.

There are documented cases where ISCCs and CSM coexist. A notable case report describes a 63-year-old male with an 11-year history of left-sided radiculopathy, ataxia, and quadriparesis. Initially, his condition was interpreted as spondylotic myelopathy with cord signal changes from the C3 to C7 levels. Despite undergoing a C3-C7 laminectomy/foraminotomy with instrumentation, the patient experienced several symptomatic recurrences. It was only after repeated magnetic resonance imaging (MRI) that a ventrally located intramedullary lesion, suspected to be a cavernoma at the C6 level, was identified. The patient eventually required surgical intervention for the cavernoma, highlighting the complexity of diagnosing and managing such cases [7]. Due to the limited evidence and lack of consensus on managing simultaneous pathological entities, this article aims to describe a specific case, outline the treatment approach, and present the postoperative results.

## Case presentation

A 66-year-old man with a past medical history of eosinophilic granulomatosis with polyangiitis presented to the emergency department with a five-month history of paresthesias in the upper extremities. Despite receiving immunosuppressive treatment for his rheumatological condition, the patient showed no clinical improvement. He also reported experiencing low back pain radiating to the right gluteal region, which he noted was causing gait impairment.

**Figure 1:**
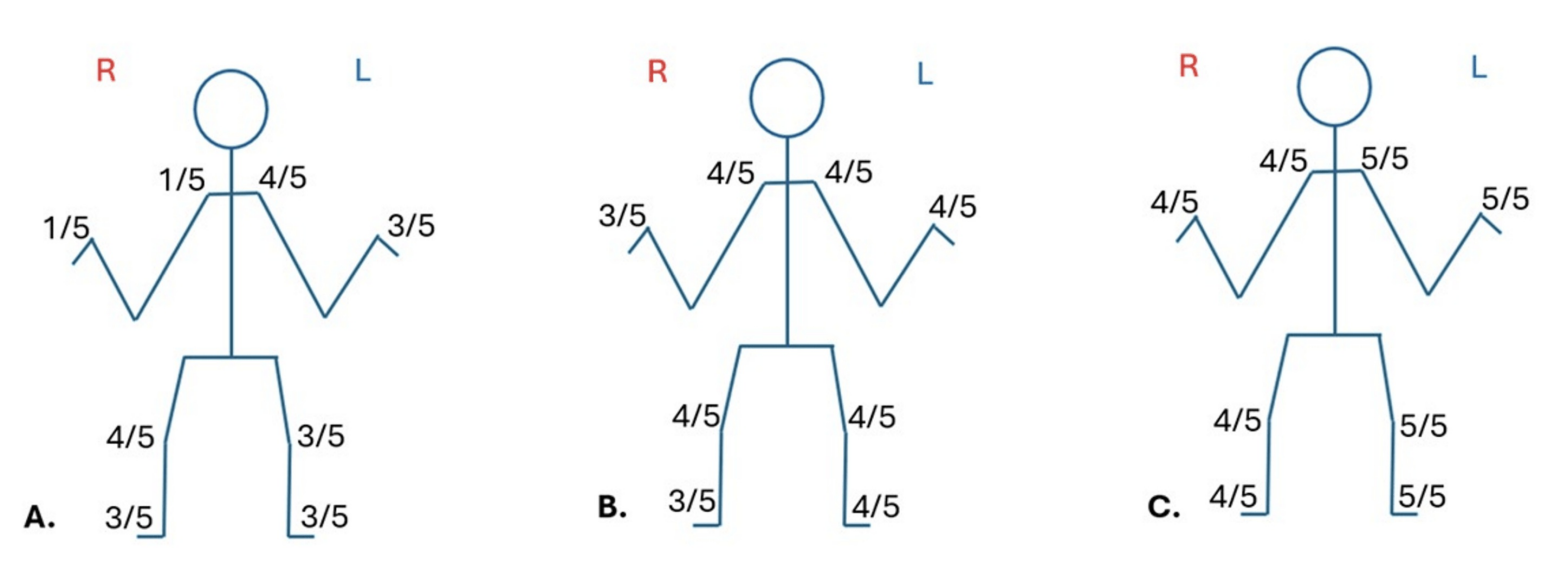
A detailed physical examination of the motor strength (A) Preoperative strength scale: the patient presented with a bilateral Babinski sign. (B) First postoperative strength evaluation (three-level C3-C6 ACDF): the patient presented with weakness accompanied by paresthesias and hyperreflexia, and a bilateral Babinski sign was present. (C) Second postoperative strength exam (cervical laminoplasty+cavernous malformation resection): the patient only presented with a right Babinski sign. ACDF: anterior cervical discectomy with cage-augmented fusion

**Figure 2:**
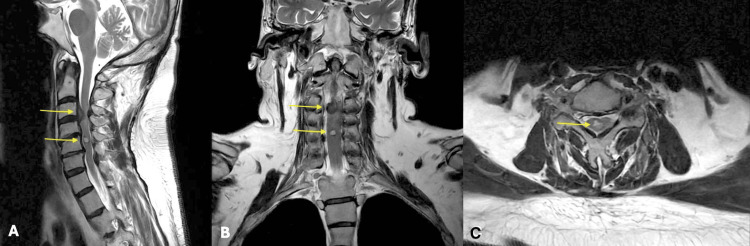
Preoperative T2-weighted MRI with sagittal, coronal, and axial reconstructions (A) Sagittal and (B) coronal T2-weighted MRI shows thickening of the posterior longitudinal ligament and disc fragment migrated into the canal of apparent C3-C4 origin. (C) Axial T2-weighted MRI shows an intramedullary lesion, central, at the level of C5, of oval morphology, heterogeneous signal, predominantly hyperintense with hypointense border, measuring 8×4×5 mm in its cephalocaudal dorsal-ventral and transverse axes, respectively (yellow arrows point to the lesions described). MRI: magnetic resonance imaging

Physical examination revealed bilateral upper motor syndrome with hypoesthesia in the lower extremities, predominantly on the right side (Figure 1A). Subsequently, an MRI was performed (Figure 2), revealing cervical spinal stenosis at C3-C4, along with an intramedullary vascular malformation lesion at C5. Differential diagnoses included cervical medullary arteriovenous malformation (AVM) vs. medullary cavernoma, among others. The final diagnosis concluded with cervical degenerative spondylotic myelopathy and a synchronous cervical medullary cavernoma.

The spine surgery service committee discussed the case due to its diagnostic difficulty and therapeutic challenges, raising the question of which entity to treat first. The decision was made to pursue staged surgical management. In the initial phase, an anterior cervical decompression through an anterior cervical discectomy with cage-augmented fusion (ACDF) was performed, followed by posterior laminoplasty with resection of the intramedullary lesion.

First stage: three-level (C3-C6) ACDF

**Figure 3:**
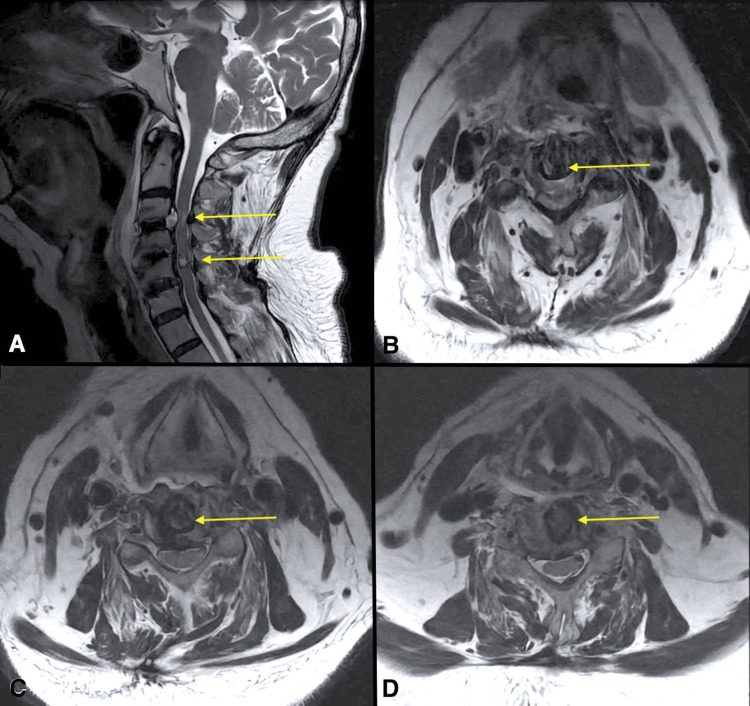
Postoperative (ACDF) T2-weighted MRI with sagittal and axial reconstructions Sagittal T2-weighted MRI showing (A) postoperative changes with intersomatic spacers C3-C4, C4-C5, and C5-C6, (B) axial postoperative changes of discectomy in C3-C4, and (C) C4-C5 and (D) C5-C6 intersomatic spaces. Central intramedullary cavernoma is observed (yellow arrows point to the lesions described). ACDF: anterior cervical discectomy with cage-augmented fusion; MRI: magnetic resonance imaging

After the surgery, the patient continued to experience predominantly weakness accompanied by paresthesias and hyperreflexia. Muscle strength powers were measured in the clinical evaluation (Figure 1B). No perioperative complications were encountered, and a follow-up MRI scan was performed (Figure 3).

Second stage: cervical laminoplasty for the resection of cavernous malformation

**Figure 4:**
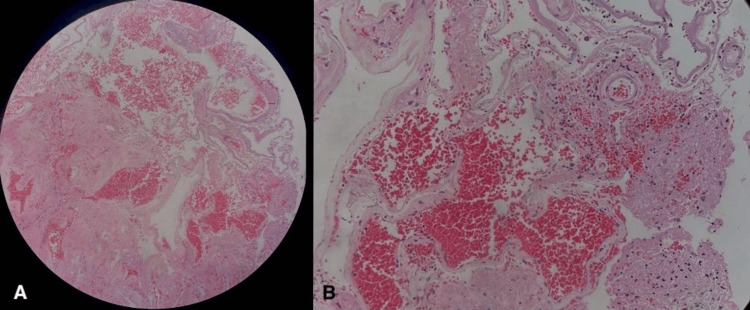
Histological features (A) H&E 40× and (B) H&E 200× show cluster lesions with abnormal and hyalinized capillaries without intervening brain tissue.

After a week of recovery, a second-stage procedure was performed, which included posterior decompression with laminectomy of C4 and C5, posterior midline spinal cord incision, cavernoma resection, and laminoplasty. During the surgery, hemosiderin deposits were found around the lesion. This finding correlated with the suspicion of a spinal cavernoma (SC). This finding correlated with the suspicion of a SC. Additionally, the histopathological study of the lesion confirmed the diagnosis, describing clusters of abnormal and hyalinized capillaries without intervening brain tissue (Figure 4).

Following the follow-up, significant clinical improvement was observed; the only remaining muscle weakness was in our patient's right upper and lower extremities (Figure 1C).

The patient's progress was favorable during a subsequent follow-up visit. He had well-controlled pain and improved motor deficits. The patient expressed satisfaction with the postoperative results.

## Discussion

ISCCs are rare vascular lesions within the spinal cord that can manifest expansive neurological manifestations depending on their location, size, and bleeding status. On the contrary, degenerative spondylotic myelopathy comprises the chronic myelopathy generated by the spinal cord compression resulting from degenerative changes in the cervical spine. To our knowledge, only one co-occurrence of CSM and intramedullary cavernomas was previously documented [8]. Managing both coexisting entities is challenging due to the overlapping symptomatology and complexity of the management approaches. Furthermore, assessing this patient's condition was further complicated by his rheumatological history. ISCC represents a significant challenge, given that the natural history of ISCC is poorly understood due to the limited evidence available in the literature, with most reports demonstrating surgical outcomes similar to brainstem cavernomas, thus advocating for similar treatment decisions: recommendation for surgical treatment after a single disabling bleed. The reported annual hemorrhage for spinal cord cavernous malformations is 2.1% (95% CI: 1.3-3.3%); it is associated with a cerebral cavernoma in 17% of the cases and a family history of cerebral cavernous malformations in 12% [9]. Significant controversy remains about whether the risk of bleeding and subsequent neurological deficit justifies the surgical treatment. The approach taken in this case and the subsequent findings can provide insights into a plausible clinical algorithm integrating the reported information on both disorders and the specific findings of this case.

Initially, it is vital to consider the diagnostic approach to each disorder, particularly the role of clinical imaging such as MRI and X-ray as an adjunctive tool to assess vertebral alignment and cervical spondylosis. For CSM, MRI is the gold standard imaging modality. However, it has limitations in documenting alignment and stability. Therefore, X-rays in vertical and dynamic positions remain essential for a comprehensive evaluation. Diagnosis is confirmed with evidence of compression, along with signs and symptoms consistent with cervical myelopathy. However, this criterion is still debated because the degree of spinal cord compression on MRI often poorly correlates with the severity of clinical presentation [10]. Due to diverse clinical manifestations, achieving a precise definition and clinical criteria for diagnosis remains challenging [11].

In contrast, cavernous malformations represent only 5-12% of spinal cord vascular lesions [12]. They typically affect females with a peak age of presentation in the fourth decade [13]. The most common locations are the thoracic (46%) and cervical segments (38%) [14]. Growth usually involves small asymptomatic bleeds that produce hemosiderin in the surrounding nerve tissue. Although the natural history is not fully understood, most cavernomas have a benign clinical course, with an annual rate of first bleeding ranging from 2.1% to 4.5% [15]. The clinical and diagnostic approach was tailored to the patient's characteristics. Deconstructing the provided information, the following key points emerge: (a) The patient, a 66-year-old man, had a history of eosinophilic granulomatosis with polyangiitis and was currently undergoing immunosuppressive therapy. (b) He presented with a five-month history of paresthesias in the upper extremities and bilateral pyramidal syndrome with hypoesthesia in the lower extremities. (c) Imaging revealed cervical spinal stenosis at the C3-C4 level and an intramedullary vascular malformation lesion at C5.

Upon careful analysis of the patient's clinical history, it becomes evident why cavernomatosis was not initially suspected, even when Mexico has a substantial population with the CCM1 gene mutation. Neither the symptoms nor the lesion's location aligns with typical cavernomatosis presentation, especially in the absence of new bleeding. Instead, they are consistent with CSM in both location and clinical characteristics. Cavernomas can bleed acutely and cause intramedullary hemorrhage, with a mass effect that can be confused with other intramedullary lesions. The clinical presentation is usually slowly progressive myelopathy, although subarachnoid hemorrhage and hematomyelia have also been reported [14].

As mentioned above, the diagnostic test of choice is the MRI with standard sequences and sensitivity-weighted images. According to the hemorrhage characteristics of MRI, sporadic ISCCs are categorized into types I-III: type I shows subacute bleeding, type II exhibits a reticulated small hematoma and thrombosis, and type III features chronic hemosiderin staining, according to the Zabramski classification [16]. Spinal cord cavernomas, like their intracranial counterparts, are not identifiable on angiography because they are low-flow lesions. The therapeutic approach comprised two phases: A three-level posterior cervical discectomy and cage-augmented fusion were performed to address the CSM, which primarily affected the C3-C4, C4-C5, and C5-C6 vertebrae. Post-surgery, the patient exhibited predominantly weakness accompanied by paresthesias and hyperreflexia. Muscle strength was assessed using the Daniels muscle testing scale, revealing various upper and lower extremity deficits. The second phase involved resection of the cavernoma detected at C5 via MRI. Notably, hemosiderin deposition around the lesion supported the suspicion of a cavernoma and the histological findings as the final diagnosis. Significant clinical improvement was observed after the interventions, with residual muscle weakness remaining in the right upper and lower extremities. The clinical outcome post-surgery and the initial suspicion guiding the approach facilitated the correlation between CSM and the cavernoma at C5 responsible for the patient's symptoms. Although the underlying cause of this syndrome remains elusive, the pathophysiological explanation may involve an initial CSM episode with minor bleeding leading to paresthesias, followed by the cavernoma causing muscle weakness and rapid deterioration. While managing CSM, the severity of myelopathy is assessed using the modified Japanese Orthopedic Association (mJOA) score, which classifies it as mild (15-17), moderate (12-14), or severe (0-11) [17].

As the literature outlines, surgical intervention is typically recommended for moderate-to-severe cases. However, the approach to mild cases remains controversial, with conservative treatment being the subject of debate due to over 50% of individuals experiencing neurological deterioration during follow-up [17,18]. Surgical considerations for electing the anterior approach initially were the lack of evidence of acute bleeding from the cavernoma, the minimal manipulation of the spinal cord required, thus reducing the risk of bleeding, achieving stabilization of the cervical spine [11], and the possibility of doing an essential decompression of the osteoligamentous components of the anterior spine. Once the anterior spine was stabilized, then we could proceed with the posterior decompression of the spinal cord by resection of the lesion. On the other hand, cavernomas are often asymptomatic lesions, leading to a recommendation for conservative management. Surgical resection becomes the preferred treatment only when symptoms manifest, as it can mitigate the associated lifelong hemorrhagic risk. On the other hand, in recent years, a new therapeutic conduct consisting of early treatment of ISCC has been adopted, with 50% of patients undergoing surgery within 32 days after presentation [19]. Patients in this cohort with paraplegia were more likely to improve at their three-month postoperative follow-up. Other studies support this principle, advocating for an increased risk of early recurrent bleeding and irreversible myelopathy secondary to mass effect [19]. Conversely, extralesional hematoma and the gliotic plane between the spinal cord tissue and the ISCC usually facilitate the resection, allowing the surgeon to resect the lesion with less trauma to the spinal tracts. In contrast to the case outlined by Dr. Nwachuku [6], as mentioned above, this patient's clinical progression followed a more insidious course, with the manifestation of muscle weakness occurring over time. While subtle discrepancies exist between these cases, they underscore the critical need for a heightened level of suspicion when encountering patients initially presenting CSM and exhibiting clinical deterioration post-surgery. In a meta-analysis performed by Fotakopoulos et al., in a pool of 396 patients, patients who have experienced a hemorrhagic episode should consider surgical intervention, which lessens the probability of recurrent hemorrhage and further neurological deficit, and surgical decompression may lead to slight neurological improvement in some patients. In non-hemorrhagic cavernomas, conservative treatment might be ideal due to the morbidity risk associated with surgery [20]. Whenever the surgical treatment is to be performed, neurophysiological monitoring of the somatosensory-evoked and motor-evoked potentials is recommended to detect approximation to sensitive and motor spinal tracts. The entry zones described for the myelotomy and resection of the cavernoma include the posterior midline entry zone, the pial entry zone, the dorsal root entry zone, and the lateral entry zone.

Managing patients with CSM and SC poses a significant clinical challenge. CSM, characterized by progressive neurological dysfunction due to cervical spine compression, and SC, a neoplastic vascular lesion, each present distinct diagnostic and therapeutic considerations.

## Conclusions

This case underscores the complexity of evaluating patients with overlapping symptoms and radiological findings. While surgery is recommended for moderate-to-severe CSM, SCs, especially asymptomatic ones, often warrant conservative management.

The findings suggest that prioritizing CSM treatment over SC reduces complications and optimizes outcomes. This requires a careful assessment of clinical presentation, imaging, and prognosis to guide treatment decisions.

Given the challenges of managing both conditions, further research and clinical experience are needed to develop standardized management strategies.
